# PReS-FINAL-2283: Systemic lupus erythematosus (SLE) in children and adolescents in pediatric unite, institute of rheumatology Belgrade, Serbia

**DOI:** 10.1186/1546-0096-11-S2-P273

**Published:** 2013-12-05

**Authors:** B Stanimirovic, G Susic, D Novakovic, R Stojanovic

**Affiliations:** 1 Clinical centar Banjaluka, Banjaluka, Bosnia and Herzegovina; 2 Institute of Rheumatology, Belgrade, Serbia

## Introduction

SLE is an autoimmune disease characterized by widespread inflammation of blood vessels and connective tissues.

## Objectives

The aim of this study is to describe the clinical and laboratory manifestations, treatment, complications and disease outcome in children and adolescents with SLE.

## Methods

Medical records of all children and adolescents with SLE treated during 10 years period (between 2001 and 2011) at the Institute of Rheumatology Belgrade were retrospectively reviewed. The collected data included informations about demographic profile, clinical and laboratory manifestations, treatment, complications and disease outcome.

## Results

Thirty seven patients (36 f, 1 m) of SLE were reviewed.. The mean age at disease onset was 15 years with a range of 7-19 years. The most common features were mucocutaneus (malar rash in 84,8%, photosensitivity in 69,7%); musculoskeletal (arthritis in 87,9%) and hematological (leucopenia in 75,8%, anemia in 69,7%, Coombs test positive in 18,9% and, thrombocytopenia in 28%). Renal involvement occurred in 45,5% of children. CNS manifestations in 51,2%. ANA was positive in 97,3%, Anti dsDNA in 81,1%, Anti Sm in 40% of our patients. Corticosteroid treatment was given in all patients in the form of prednisone (100%) and methylprednisolone, iv pulses were applied in 58,8%. Antimalarics were used in 97.3%, azathiophrine in 48,6%, micophenolat-mofetil in 21,6% and cyclosporine in 3,8% of children. The most common complications were hypertension, hypercorticism and opportunistic infections. Three patients died during the period of the study, two girls according to antiphospholipid syndrome complications, one of infection (sepsis).

## Conclusion

The most common features were mucocutaneus, musculosceletal and hematological. Less than half of the patients were with renal involvement, although 80% were anti ds DNA positive. All patients were treated with corticosteroids, and except three with antimalarics. High blood pressure, hypercorticism and opportunistic infections were most common complications. There was no significant difference in clinical and laboratory manifestations, therapy approach and outcome compared to those in most pediatric SLE studies. Low number of patients with renal involvement can be explained by profile of institution.

## Disclosure of interest

None declared.

